# Quantitative Histomorphometric Features of Prostate Cancer Predict Patients Who Biochemically Recur Following Prostatectomy

**DOI:** 10.1016/j.labinv.2023.100269

**Published:** 2023-10-26

**Authors:** Savannah R. Duenweg, Michael Brehler, Allison K. Lowman, Samuel A. Bobholz, Fitzgerald Kyereme, Aleksandra Winiarz, Biprojit Nath, Kenneth A. Iczkowski, Kenneth M. Jacobsohn, Peter S. LaViolette

**Affiliations:** a Departments of Biophysics, Medical College of Wisconsin, Milwaukee, Wisconsin; b Radiology, Medical College of Wisconsin, Milwaukee, Wisconsin; c Pathology, Medical College of Wisconsin, Milwaukee, Wisconsin; d Urology, Medical College of Wisconsin, Milwaukee, Wisconsin; e Biomedical Engineering, Medical College of Wisconsin, Milwaukee, Wisconsin

**Keywords:** annotations, digital pathology, image processing, pathomic features, prostate cancer, whole slide images

## Abstract

Prostate cancer is the most commonly diagnosed cancer in men, accounting for 27% of the new male cancer diagnoses in 2022. If organ-confined, removal of the prostate through radical prostatectomy is considered curative; however, distant metastases may occur, resulting in a poor patient prognosis. This study sought to determine whether quantitative pathomic features of prostate cancer differ in patients who biochemically experience biological recurrence after surgery. Whole-mount prostate histology from 78 patients was analyzed for this study. In total, 614 slides were hematoxylin and eosin stained and digitized to produce whole slide images (WSI). Regions of differing Gleason patterns were digitally annotated by a genitourinary fellowship-trained pathologist, and high-resolution tiles were extracted from each annotated region of interest for further analysis. Individual glands within the prostate were identified using automated image processing algorithms, and histomorphometric features were calculated on a per-tile basis and across WSI and averaged by patients. Tiles were organized into cancer and benign tissues. Logistic regression models were fit to assess the predictive value of the calculated pathomic features across tile groups and WSI; additionally, models using clinical information were used for comparisons. Logistic regression classified each pathomic feature model at accuracies >80% with areas under the curve of 0.82, 0.76, 0.75, and 0.72 for all tiles, cancer only, noncancer only, and across WSI. This was comparable with standard clinical information, Gleason Grade Groups, and CAPRA score, which achieved similar accuracies but areas under the curve of 0.80, 0.77, and 0.70, respectively. This study demonstrates that the use of quantitative pathomic features calculated from digital histology of prostate cancer may provide clinicians with additional information beyond the traditional qualitative pathologist assessment. Further research is warranted to determine possible inclusion in treatment guidance.

## Introduction

Prostate cancer (PCa) accounts for approximately one in 4 new male cancer diagnoses, making it the most commonly diagnosed cancer in American men. An estimated 268,000 new cases of PCa will be diagnosed in 2022 in the United States alone, although not all cases are at high risk for metastasis or death.^1^ Improved prostate cancer screening and treatments have led to a high 5-year overall survival rate; however, despite this, between 20% and 30% of patients with prostate cancer will experience recurrence within 5 years of therapy.^2–4^ There has been a growing interest in pathological studies for developing improved strategies for predicting and preventing the biochemical recurrence of PCa.

Prostate cancer is currently diagnosed using the Gleason grading scale, which assigns a score corresponding to the Gleason grades of the 2 most predominant cell patterns on histopathological assessment. More recently, these patterns have been used to assign patients into 5 Grade Groups (GG) to predict prognosis.^1^ Low-risk diseases may be managed through active surveillance, whereas clinically significant cancer (GG ≥2, tumor volume ≥0.5 mL, or stage ≥T3) is often treated with therapies such as radical prostatectomy or radiation. Prostatectomy is considered curative if tumors are organ-confined; however, distant metastases may form and result in biochemical recurrence (BCR).

Biochemical PCa recurrence is determined by increasing prostate-specific antigen (PSA) scores after treatment. However, PSA is currently the only validated biomarker for disease recurrence.^2^ After surgery, PSA levels should drop to 0 ng/mL. Typically, the cancer is considered recurrent when PSA levels rise above 0.1–0.2 ng/mL, although some physicians use higher PSA thresholds or wait to observe the elevated PSA score on 2 or more successive tests.^3,4^

In recent years, whole slide images (WSI), resulting from the use of microscopes for digitizing glass slides with histology samples, have become increasingly popular. Digital pathology enables fast acquisition, management, and interpretation of histology.^5^ Additional opportunities have arisen from the use of digital pathology, such as pathological workflows including computational algorithms and applications of artificial intelligence.^6^ Quantitative histomorphometric approaches to define the features of tumor morphology have previously discriminated between the gland and nuclear shape and architectural and textural features between Gleason grades in prostate cancer histology.^7,8^ Additionally, recent studies have shown that in benign and less aggressive prostate cancers, local gland orientations are similar to each other, but these orientations differ in aggressive diseases; thus, gland angularity was able to predict PCa BCR.^9^

These recent studies, although promising, were limited to tissue microarrays, needle biopsies, or small sections of WSI. Additionally, the process of assigning Gleason grades is subjective and relies on manual grading by experienced pathologists, which can be time-consuming and result in interobserver variability between regions of high and low-grade cancers.^6^ This study aims to determine whether quantitative pathomic features of prostate cancer differ in patients who exhibit biochemical recurrence after surgery using WSI and high-resolution tiles taken from pathologist-annotated cancer and noncancer regions. Specifically, we tested the hypothesis that histomorphometric features calculated from digital pathology would be able to predict BCR better than clinicopathological features alone.

## Materials and Methods

### Patient Population and Data Acquisition

Data from 78 prospectively recruited patients with biopsy-confirmed PCa undergoing radical prostatectomy between 2014 and 2021 were analyzed for this institutional review board-approved study. Patients underwent multiparametric magnetic resonance imaging before prostatectomy on a 3T magnetic resonance imaging scanner (General Electric or Siemens Healthineers) using an endorectal coil. Each protocol included T2-weighted imaging, dynamic contrast-enhanced imaging, and diffusion-weighted imaging.

After surgery, patients were monitored with PSA testing according to the standard-of-care practices. Patients were followed up for more than 7 years after surgery, and those with a measured PSA score of at least 0.2 ng/mL at any time point after surgery were considered biochemically recurrent. Inclusion criteria for this study included digitized and annotated histology after radical prostatectomy and at least one postsurgery PSA test. Additionally, patients were excluded if digitized histology was of poor quality (ie, rips, tears, low resolution, etc.). Clinicopathological features and demographic information for the study cohort are summarized in Table 1.

### Histopathologic Analysis

**Figure 1.**
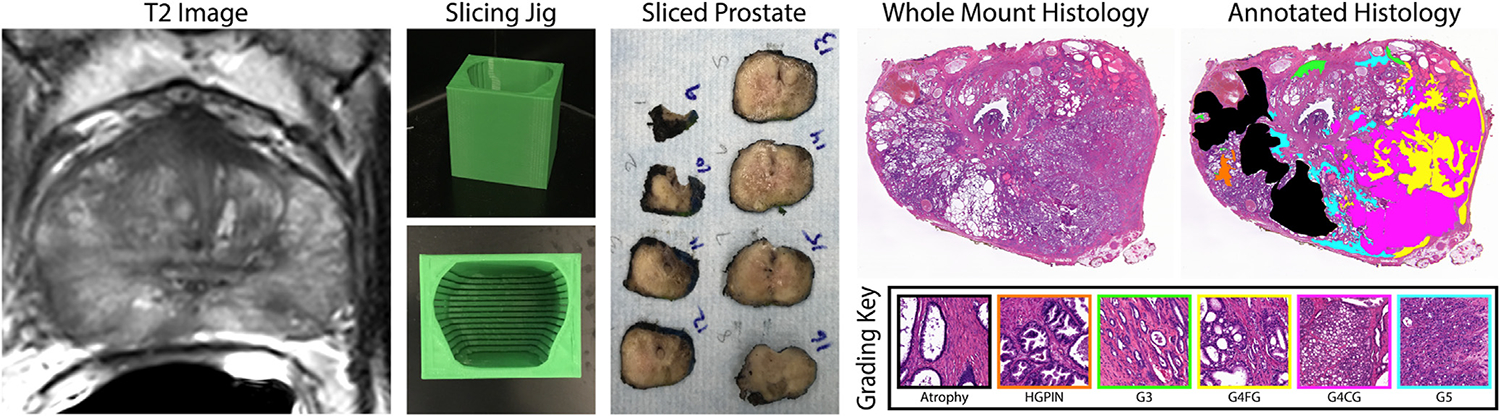
Schematic representation of the annotation and tile extraction process, with second-order feature segmentations across a WSI. Top left: T2-weighted MR image used to model the prostate slicing jig. Custom prostate slicing jigs allow the prostate to be sliced to match the slice thicknesses of the MR image. Top right: whole-mount samples were stained, digitized, and annotated by a pathologist. Annotations were color-coded by class to extract representative tiles from each of the annotation classes: atrophy, HGPIN, G3, G4NC, G4CG, G5, and Seminal Vesicles (not pictured). Bottom: pathomic features are calculated across WSI and feature maps are overlaid on the original image.

Robotic prostatectomy was performed approximately 2 weeks after imaging using the da Vinci system (Intuitive Surgical).^10,11^ Surgical margin status was not explicitly reported in our patient notes; however, surgical stage, which factors in margins, was included in our analyses. Prostate samples were fixed in formalin overnight and sectioned using custom slicing jigs.^12^ Prostate masks used to create each patient-specific slicing jig were manually segmented from the patient’s T2-weighted image using Analysis of Functional NeuroImages (http://afni.nimh.nih.gov/),^13^ 3D modeled using 3dSlicer (slicer.org), and imported into Blender 2.75 (https://www.blender.org/) to digitally extract from a universal slicing jig to match the orientation and slice thickness of each patient’s T2-weighted image.^14–17^ The patient-specific jigs were then 3D printed using a fifth-generation Makerbot (Makerbot Industries; Fig.1).

Whole-mount tissue sections were paraffin-embedded, sectioned, and hematoxylin and eosin (H&E)-stained in our histology core laboratory. The stained slides were digitally scanned at 40× magnification using either an Olympus (Olympus Corporation) (*n* = 47 patients, 359 slides) or a Huron (Huron Digital Pathology; *n* = 32 patients, 256 slides) sliding stage microscope at resolutions of 0.34 or 0.2 microns per pixel, respectively. Due to the large file size that these images had, downsample factors of 8 and 10 for the Olympus and Huron slide scanners were applied, respectively—where the Huron scanner used a larger downsampling factor due to the increased resolution (ie, magnification of 5 or 4, and resolution of 0.04 or 0.02 microns per pixel, respectively). Two slide scanners were used in this analysis due to a recent equipment upgrade. Although this may be a confounding factor in our analyses, we opted to use a greater number of patients and slides rather than limit ourselves to less available data. Slides were downsampled for computational processing, ensuring a comparable downsampling factor between the 2 slide scanners. A total of 614 digitized slides across all patients (mean: 7.9 slides, range: 2–14 per patient) were manually annotated for different Gleason patterns by a genitourinary fellowship-trained pathologist using a stylus on Microsoft Surface Pro 4 (Microsoft) with a preloaded color palette. Annotations from 289 slides were performed on digital images produced on a third lower resolution slide scanner at 40× magnification and 0.85 μm/px (Nikon Metrology). These slides were rescanned, and the annotations were brought into the higher resolution space of the previously aforementioned slide scanners. A flowchart for the slide scanners used and their respective properties can be found in Supplementary Figure S1. The manually annotated tissue classes include seminal vesicles, atrophy, high-grade prostatic intraepithelial neoplasia (HGPIN), Gleason 3 (G3), Gleason 4 non-cribriform glands (NC), Gleason 4 cribriform (to papillary) glands (CG), and Gleason 5 (G5). Analysis was performed on G4CG and G4NC separately as there are notable prognostic differences between the two.^18–22^ However, G4CG was defined as true papillary, small cribriform (ie, rounded acinar spaces with ≤12 lumens and no solid area), and large cribriform (ie, more sprawling, cribriform to focally solid formations). An example slide with annotations can be found in Figure 1.

### Annotation Segmentation

Digital whole-mount mount slides were divided into high-resolution tiles that were 3,000 × 3,000 pixels or 5,100 × 5,100 pixels for the Olympus or Huron microscopes, respectively. The Olympus scanner was originally used for slide scanning, and a tile size of 3,000 × 3,000 pixels was previously found to be sufficient for showing gland morphology. When upgrading to the use of the higher resolution Huron scanner, a larger tile size was chosen to capture a comparable amount of histology per tile. The tiles were named using xy-coordinates corresponding to their location within the WSI. These tiles were stitched back together to recreate the WSI and concurrently create x- and y-coordinate look-up tables.

Annotations previously performed on the lower resolution microscope were aligned to high-resolution WSI using the MATLAB R2021b’s (The MathWorks) *imregister* function. Each of the possible annotations was isolated into individual masks, including a mask for nonatrophic benign tissues. An additional averaged white image of nontissue was found to remove the background or primarily white spaces from the masks. Annotation masks were divided into regions of interests (ROIs) per lesion. Therefore, ROIs were individually compared with the xy-look-up tables to determine coordinates corresponding to tiles within the ROI. Five randomly selected tiles that were considered more than 50% within the mask after white-space removal were saved into annotation-specific directories. For nonatrophic benign tissues, 15 tiles were selected instead to not only obtain the most representative examples of benign tissue but also a balance between cancer and benign tissue tiles. Additionally, ROIs that were too small to extract 5 tiles from were skipped. A representative tile from each annotation mask is shown in Figure 1. Table 2 breaks down the distribution of patients, slides, and Gleason-annotated histology tiles per slide scanner.

### Pathomic Feature Calculation

**Figure 2.**
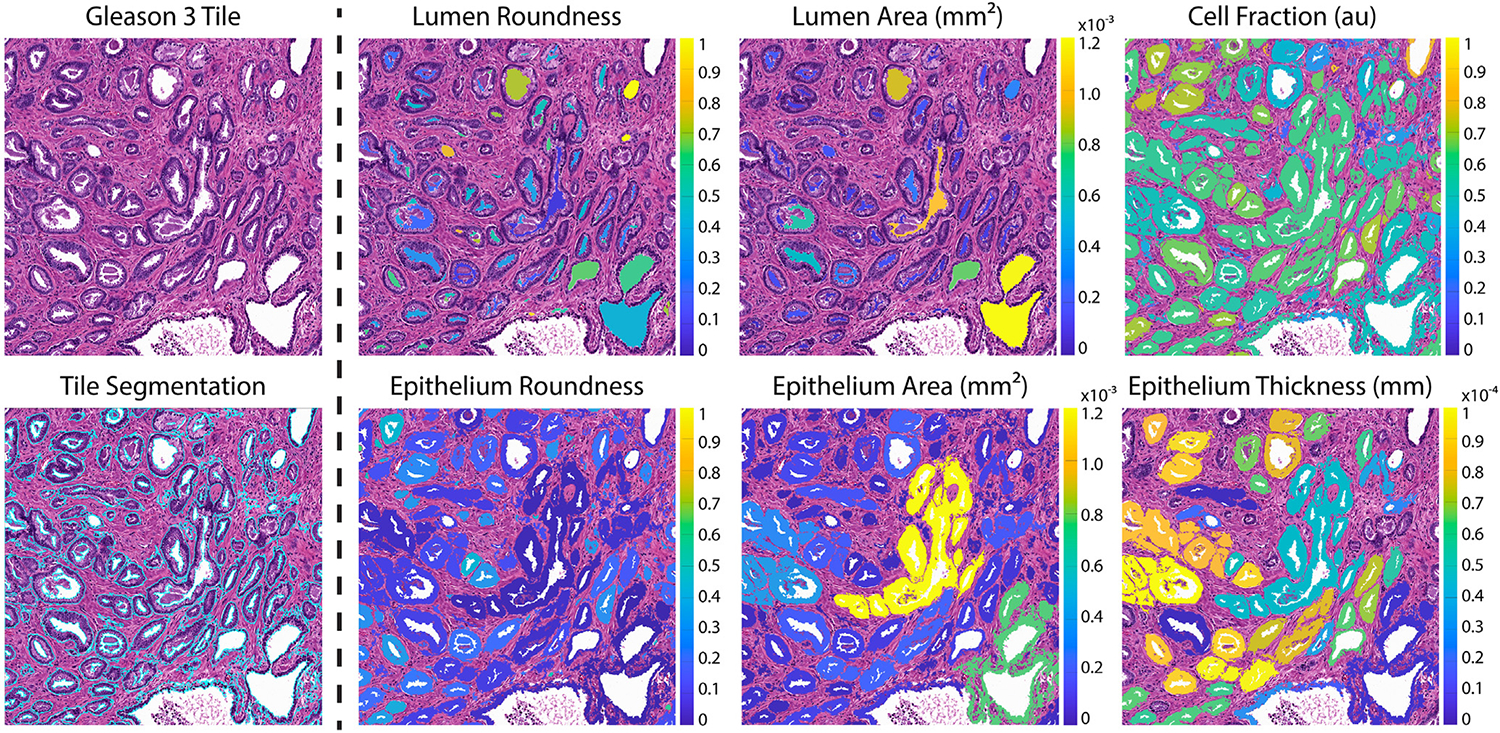
Pathomic feature segmentations. A representative Gleason 3 tile from the Huron microscope with pathomic feature maps. Calculated features include lumen roundness and area; cell fraction; epithelial roundness, area, and wall thickness. Calculated values are overlaid on the respective glands. Units of area maps are in mm^2^, and thickness in mm. Roundness and cell fraction are unitless.

Both high-resolution tiles and WSI were first downsampled by a factor of 2 to decrease the processing time while maintaining the highest resolution. Images were then processed using custom, in-house MATLAB (Mathworks Inc) pipelines to extract pathological features for quantitative analysis. A color deconvolution algorithm was first applied to segment stroma, epithelium, and lumen based on their corresponding stain optical densities (ie, positive hematoxylin or eosin and background),^23^ which visually proved to create better histology masks than using RGB color channels. These features were then filtered and smoothed to remove noise and improve histology segmentations using built-in MATLAB functions from the *Image Processing Toolbox*. These resulting masks were used to automatically calculate histomorphometric features per gland, including lumen roundness and area; epithelial roundness, area, and wall thickness; and cell fraction (ie, the percent of epithelial cells per total gland area, defined by the area of the epithelium without lumen). Individual lumen and epithelium were numbered using *bwlabel*, perimeters were defined using *bwboundaries*, and areas were calculated using *regionprops*. Epithelial wall thickness was defined as the minimum distance between the inner and outer edges of the gland. Roundness was calculated using the following equation: $\frac{4\pi Area}{Permeter^{2}}$. Additionally, overall stromal and epithelial areas were computed using *bwarea* and normalized to the image area as the tile sizes differed between the 2 microscopes (ie, 3,000 × 3,000 pixels and 5,100 × 5,100 pixels) and variation in prostate size across WSI. Figure 2 shows an example of the pathomic feature calculations across an extracted tile from the Huron scanner. An example WSI from the Huron scanner is shown in Figure 1, and one from the Olympus scanner is demonstrated in Supplementary Figure S2.

### Tumor Volume Calculation

In addition to pathomic features, we also calculated the tumor volume and ratio for each patient for additional quantitative information to the model. The patient-specific prostate masks previously described were used to calculate the patient’s prostate volume by multiplying the number of voxels present in the mask by the size in the x-, y-, and z-planes. However, WSI was segmented into their individual annotation classes, and the area of each annotation was calculated, as well as the total tissue area per slide. The total area of cancerous regions was summed and normalized to the total tissue area per slide to determine the ratio of cancer to noncancer on a slide. This ratio was then multiplied by the patient’s prostate volume to approximate the volume of tumor present.

### Statistical Analysis

To evaluate the ability of pathomic features to predict biochemical recurrence, two-tailed, two-sample *t* tests were used to determine whether individual pathomic features were significantly different between those who did or did not experience biochemical recurrence across the 3 tile groups and WSI. Logistic regressions were fit to assess the ability of the pathomic features, as well as tumor volume and cancer ratio, to predict BCR (PSA ≥0.2 ng/mL postsurgery) using both features calculated across tiles and WSI using SPSS Statistics (IBM, Armani). From the tile-level analysis, a model was fit per tissue type (all tiles, cancer only, and noncancer only). Additionally, models were fit using clinicopathological information, which included age, surgery stage, Grade Group, pre-surgery PSA, tumor ratio/volume, CAPRA score, and Grade Group alone to compare current gold standard predictors of BCR to pathomic features. Finally, 2 models encompassing all tile pathomic features or WSI pathomic features with clinicopathological features were fitted to determine whether all features together could further predict recurrence. Multiple comparison corrections were performed using the Benjamini–Hochberg false discovery rate (FDR) procedure.^24^ All logistic regression models were evaluated for performance using receiver operator characteristic (ROC) curves, and performance was quantified by area under the curve (AUC). Kaplan–Meier survival curves were generated on the all-tile pathomic feature group, cribriform presence,^19,20,25^ CAPRA score,^26^ and Gleason Grade Groups to directly visualize observed survival and determine significance; however, a Cox proportional hazards regression was used to generate hazard ratios. Tile pathomic features were divided into low and high risk for recurrence using a threshold of 0.2 based on the ROC curve. Additionally, CAPRA scores were merged into low, medium, and high risk for recurrence.^27^

## Results

**Figure 3.**
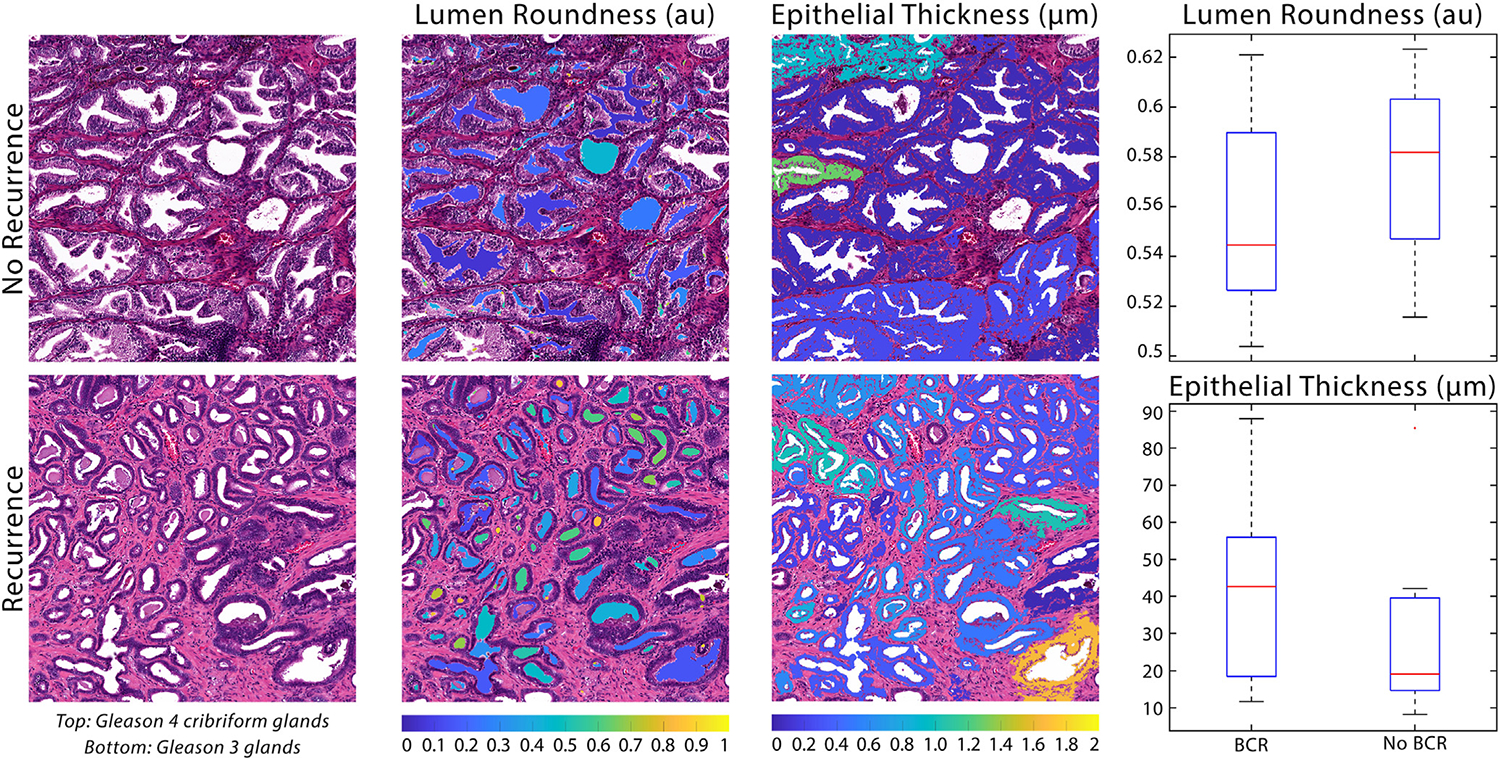
Significant feature predictors of BCR with examples from cancer tiles. Top left: the patient who did not experience biochemical recurrence had regions of papillary to cribriform glands that had been previously associated with BCR. Bottom left: the patient who did experience BCR had regions of low-risk G3 cancer. Middle: feature maps overlaid on tiles.

From our *t* test analyses of independent pathomic features, across all tile groups and WSI, lumen roundness was found to be significantly different between those who did and did not experience BCR (all *P* <.05). Additionally, epithelial wall thickness was significantly different in the cancer-only tile group (*P* <.05). Features that we found to be significant predictors of BCR from the logistic regressions were further examined to determine the relationship between the feature and BCR. Figure 3 compares representative cancer tiles from 2 patients who did or did not develop biochemical recurrence.

**Figure 4.**
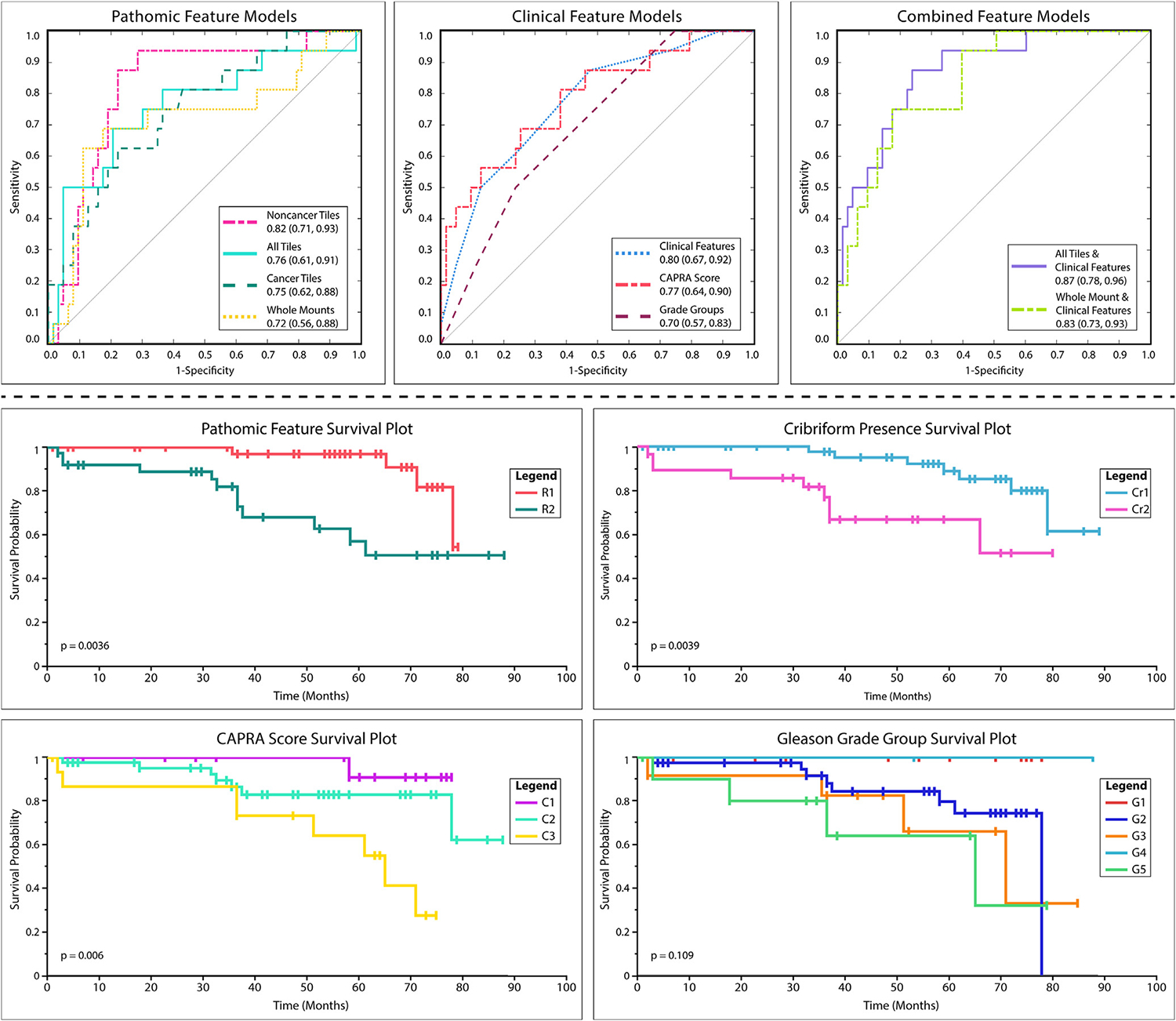
Top: ROC curves for each logistic regression model with AUCs and 95% CI. Pathomic feature models were generated for noncancer, cancer, all tiles, and WSI. Clinical feature models included a general model encompassing clinicopathological information, CAPRA score, and Grade Groups. Combined feature models included tile or WSI pathomic features with clinicopathological information. Bottom: Kaplan–Meier survival analyses were conducted to compare survival of pathomic features, cribriform presence, CAPRA score, and Gleason Grade Groups (*P* values calculated using log-rank test). In our patient cohort, higher grade cancers were not significantly more likely to recur. R1, low-risk; R2, high-risk; Cr1, cribriform glands absent; Cr2, cribriform glands present; C1, CAPRA 0–2; C2, CAPRA 3–5; C3, CAPRA 6–10; G1–5, Gleason Grade Group 1–5.

Logistic regression classified each of the tile groups at accuracies of 86%, 84%, and 81% for all tiles, cancer, and noncancer, respectively, and area under the ROC curve of >0.70 for the all tiles and cancer groups and 0.82 for the noncancer group. The WSI model performed at an accuracy of 84% with an AUC of 0.72. These were comparable with our clinical feature models that had accuracies of 81%, 86%, and 80% for encompassing clinical features, CAPRA score, and GG only, respectively, with AUCs of 0.78, 0.77, and 0.70. Finally, the models encompassing pathomic and clinical features performed at accuracies of 86% and 84% with AUCs of 0.78 and 0.82 for tile or WSI features, respectively. ROC curves comparing pathomic feature, clinical, and combined models are shown in Figure 4, top. Each model was statistically compared using a randomized permutation test (*n* = 10,000 permutations; Table 3), and the pathomic feature model had a statistically significant higher AUC compared with the 3 clinical feature models (all *P* <.01) and a smaller AUC compared with both combined models (both *P* <.05). Additionally, the combined feature models statistically outperformed all 3 of the clinical feature models (all *P* <.005).

The Kaplan–Meier analyses, using the log-rank test to determine significance, revealed that no significant differences in time to recurrence existed between Gleason Grade Groups (*P* =.1; Fig. 4, bottom). Although we found our logistic regression models were comparable across pathomic and clinical feature models, we found that the Kaplan–Meier survival analysis using pathomic features marginally outperformed CAPRA score and cribriform presence; however, all 3 models showed significant time differences to recurrence (*P* =.0036,.006, and.0039, respectively). Individual hazard ratios for features within models can be found in Table 4. Note that the CAPRA and Grade Group models needed to be simplified for model convergence. Additionally, CAPRA (1) includes scores 0–2, CAPRA (2) includes scores 3–5, and the reference class includes scores 6–10. Similarly, Grade Group (1) includes GG1 and 2, Grade Group (2) includes GG3, and the reference class includes GG4 and 5.

Table 5 shows b values and *P* values for each feature within each model, where *P* values indicate the significance of individual features between recurrent and nonrecurrent groups, stratified through our logistic regression models. After FDR correction, these results did not maintain significance, indicating that although no individual feature may predict biochemical recurrence, the combination of features can. Regression tables for the clinical features, Grade Group only, and combined pathomic and clinical feature models can be found in Supplementary Tables S1 and S2.

## Discussion

The high rate of prostate cancer recurrence has led to growing interest in predicting biochemical recurrence to identify individuals at high risk for adverse outcomes early and accurately. Of the 78 patients in this study cohort, 16 experienced eventual BCR. This study showed that the use of quantitative pathomic features calculated from digital histology of PCa, as well as tumor volume and ratio, may provide clinicians with better information than the traditional qualitative pathological assessment. Most notably, we show that in areas of noncancer, pathomic features can predict BCR better than Gleason Grade Groups and CAPRA score, indicating that gland morphologies differ in noncancer regions between patients who will recur and those that will not.

The likelihood of recurrence is typically thought to increase depending on the aggressiveness of the cancer, as defined by the Gleason Grade Groups, the current gold standard prognostic indicator of BCR; however, this system is subjective and prone to significant interobserver variability.^17,28^ The Gleason Grade Group model (ROC AUC = 0.70, *P* =.1) performed worse than all pathomic features models, as well as the CAPRA and cumulative clinical feature models (ROC AUC range: 0.72–0.87, *P* <.05). Of note, 7 patients in our cohort graded as GG5, with the worst prognosis, did not experience a recurrence, indicating room for improvement in the current system. Quantitative features of prostate cancer histology may aid clinicians in predicting a patient’s risk of biochemical recurrence compared with a qualitative analysis of gland morphology. This may be especially highlighted due to the increased performance of cribriform presence survival analysis compared with Grade Groups, which has an obvious difference in gland morphology compared with other patterns.

Machine and deep-learning approaches are becoming increasingly popular in digital pathology studies, especially regarding automated grading of cancers to reduce the time burden on pathologists. Machine learning algorithms use computationally derived metrics to assess information similarly to human intervention, whereas deep-learning models use deeper features that are extracted throughout the process and eliminate human intervention. A previous deep-learning study showed luminal features to be prognostic of BCR.^29^ We showed that in addition to luminal features, epithelial and stromal features can also stratify patients by BCR risk. A recent machine learning study applied a model to 16 clinical features to predict BCR.^30^ This model achieved an accuracy of 97%; however, we show in our analyses that adding quantitative pathomic features to clinicopathological reports could further improve predictive power.

In this study, we tested the hypothesis that pathomic features of prostate cancer can predict patients who eventually exhibit biochemical recurrence after radical prostatectomy. Of the 8 features calculated with our pathomic feature calculator, lumen roundness was found to be a significant predictor of BCR in all pathomic feature models. Epithelial wall thickness was significantly different within the cancer tile group. These findings suggest that the variation in gland architecture across tiles and WSI may be related to prostate cancer aggressiveness.

Although the results of this study are promising, several limitations exist to note. This study uses a relatively small patient cohort compared with previous prostate cancer studies; thus, a larger cohort of patients may show different pathological characteristics. The small number of patients in this cohort who experienced eventual biochemical recurrence limited our ability to create a test dataset or perform multiple comparison corrections to assess our models’ performances thoroughly. Future studies assessing these features as predictors of BCR should look to larger datasets with greater representation of BCR to fully evaluate these pathomic features. Additionally, this was a single-center study with one pathologist’s annotations of the data. Future studies may seek external validation and comparison of additional pathologist annotations to increase the generalizability of the models. Finally, a subset of our annotations was completed on whole-mount samples that were digitized using lower resolution sliding stage microscope and were later rescanned at a higher resolution. Efforts to align and rescale the low-resolution annotations to a higher resolution may have introduced minor alignment differences.

We demonstrate in a cohort of 78 patients who underwent surgery for prostate cancer that those who had an increase in lumen roundness by prostate histology showed an increased risk for biochemical recurrence after prostatectomy. The use of quantitative histomorphometric feature models calculated from the digital histology of PCa was comparable with the traditional qualitative classification of patients defined by clinicopathological information and Gleason Grade Groups alone. Furthermore, combining the quantitative features with standard clinical features performed best, indicating that the addition of pathomic features is superior to the information from the qualitative reports alone. Further research is warranted to determine possible inclusion in treatment guidance. Additional studies should probe the inclusion of machine and deep-learning applications to further predict the risk of recurrence.

## Supplementary Material

Supplemental Doc

## Figures and Tables

**Table 1:** Summary of clinicopathological features of the prostate cancer cohort at the time of radical prostatectomy

	Recurrence	Nonrecurrence	Total
	(*n* = 16)	(*n* = 63)	(*n* = 79)

Age at RP, years (mean, SD)	58 (7.3)	59 (6.2)	59 (6.4)
Race (*n*, %) (*n* = 77)			
African American	3 (25)	9 (75)	12 (15)
White/Caucasian	12 (18)	53 (82)	65 (83)
Asian	1 (100)	0 (0)	1 (1)
Other	0 (0)	1 (100)	1 (1)
Preoperative PSA, ng/mL (*n*, %)			
<6	5 (12)	36 (88)	41 (52)
≥6–10	4 (16)	21 (84)	25 (32)
≥10–20	6 (55)	5 (45)	11 (14)
≥20–30	1 (50)	1 (50)	2 (2)
Grade group at RP (*n*, %)			
6	0 (0)	14 (100)	14 (18)
3 + 4	8 (20)	32 (80)	40 (51)
4 + 3	4 (33)	8 (67)	12 (15)
8	0 (0)	2 (100)	2 (2)
≥9	4 (36)	7 (64)	11 (14)
Clinical stage (*n*, %)			
T1	12 (18)	53 (82)	65 (82)
T2	4 (29)	10 (71)	14 (18)
Surgical stage (*n*, %)			
2a,b	0 (0)	7 (100)	7 (9)
2c	7 (17)	35 (83)	42 (53)
3a,b	9 (30)	21 (70)	30 (38)
Cribriform presence (*n*, %)			
Present	9 (33)	18 (67)	27 (34)
Not present	7 (13)	45 (87)	52 (66)
Follow-up time after RP, years (mean, range)			4.1 (0.1–7.3)
Time to BCR, years (mean, range; *n* = 21)		2.5 (0. 13–5.9)	

BCR, biochemical recurrence; PSA, prostate-specific antigen; RP, radical prostatectomy.

**Table 2:** Breakdown of patients, slides, and tiles per scanner

	Olympus	Huron	Total

Patients (*n*, %)	47 (59)	31 (41)	78
Glass slides (*n*, %)	359 (58)	255 (42)	614
Annotated tiles (*n*, %)			
Atrophy	5,973	2,174	8,147 (36)
G3	1,495	830	2,325 (10)
G4CG	310	205	515 (2)
G4NC	740	565	1,305 (6)
G5	99	114	213(1)
HGPIN	340	0	340(1)
Seminal vesicles	728	145	873 (4)
Unlabeled tissue	5,372	3,819	9,191 (40)
**Total/scanner**	15,057	7,852	22,909 (100)
Glass slides per patient (mean, range)			7.9 (2–14)
Annotation classes per patient (mean, range)			4.9 (3–8)
Tiles per patient (mean, range)			293.7 (90–765)

**Table 3:** Randomized permutation test results comparing statistical differences between each tested model

Model 1	Model 2	P-value

Pathomic features	Clinical features	.009
	CAPRA	.009
	Grade groups	<.001
	All tiles and clinical features	.014
	WSI and clinical features	.06
Clinical features	CAPRA	.45
	Grade groups	.18
	All tiles and clinical features	<.001
	WSI and clinical features	<.001
CAPRA	Grade groups	.36
	All tiles and clinical features	.003
	Whole mounts and clinical features	.003
Grade groups	All tiles and clinical features	<.001
	WSI and clinical features	<.001

**Table 4:** Kaplan—Meier and Cox proportional hazards regression models to determine the relative risk of eventual biochemical recurrence

Feature	*P*-value	Hazard ratio	95% CI

Pathomic features	.004*	2.23	1.68, 2.95
Cribriform	.004*	3.40	1.46, 3.41
CAPRA	.006*		-
CAPRA (1)	.30	3.02	0.37, 24.67
CAPRA (2)	.01	14.27	1.77, 115.19
Grade group	.11*		-
Grade group (1)	.13	2.52	0.75, 8.43
Grade group (2)	.15	2.48	0.73, 8.43

Hazard Ratios and within group P values were calculated using the Cox proportional hazards model and corresponding Wald test. The 4 main groups’ significance was determined using KaplaneMeier survival analysis and corresponding log-rank test (denoted with an *).

**Table 5:** Logistic regression feature importance from pathomic feature models stratified by tile group and WSI

Group	Feature	β (SE)	*P*-value

All tiles	Tumor ratio (au)	−18.23 (9.67)	.06
	Tumor volume (mm^3^)	0.49 (0.25)	.05
	Stroma area (mm^2^)	47.1 (83.36)	.57
	Epithelial area (mm^2^)	20.89 (90.07)	.82
	Epithelial size (mm^2^)	63.33 (226.71)	.78
	Epithelial roundness (au)	25.24 (46.34)	.59
	Epithelial wall thickness (mm)	3652.68 (7327.77)	.62
	Cell fraction (au)	−236.05 (575.2)	.68
	Lumen area (mm^2^)	5.85 (7.25)	.42
	Lumen roundness (au)	32.18 (23.63)	.17
Cancer	Tumor ratio (au)	−15.45 (8.51)	.07
	Tumor volume (mm^3^)	0.4 (0.22)	.08
	Stroma area (mm^2^)	51.27 (46.34)	.27
	Epithelial area (mm^2^)	45.67 (57.36)	.43
	Epithelial size (mm^2^)	34.05 (108.86)	.75
	Epithelial roundness (au)	16.43 (25)	.51
	Epithelial wall thickness (mm)	−110.79 (313.09)	.72
	Cell fraction (au)	−5.6 (4.31)	.19
	Lumen area (mm^2^)	−2191.48 (8417.29)	.8
	Lumen roundness (au)	26.54 (15.34)	.08
Noncancer	Tumor ratio (au)	−13.75 (8.69)	.11
	Tumor volume (mm^3^)	0.4 (0.24)	.09
	Stroma area (mm^2^)	−8.37 (68.69)	.9
	Epithelial area (mm^2^)	−109.4 (117.55)	.35
	Epithelial size (mm^2^)	−49.6 (220.67)	.82
	Epithelial roundness (au)	0.78 (55.73)	.99
	Epithelial wall thickness (mm)	152.44 (514.12)	.77
	Cell fraction (au)	7.81 (7.7)	.31
	Lumen area (mm^2^)	−604.46 (5544.68)	.91
	Lumen roundness (au)	37.85 (24.79)	.13
Whole slide image	Tumor ratio (au)	−14.99 (8.47)	.08
	Tumor volume (mm3)	0.38 (0.22)	.08
	Stroma area (mm^2^)	74.93 (58.42)	.2
	Epithelial area (mm^2^)	1.23 (45.95)	.98
	Epithelial size (mm^2^)	−0.02 (0.02)	.52
	Epithelial roundness (au)	3.57 (15.68)	.82
	Epithelial wall thickness (mm)	3.91 (3.77)	.3
	Cell fraction (au)	1.76 (8.08)	.83
	Lumen area (mm^2^)	32.08 (102.23)	.75
	Lumen roundness (au)	6.1 (5.08)	.23

β-values are shown with standard error (SE). P values are presented uncorrected, but α was correct using FDR. Degrees of freedom = 1.

## Data Availability

The data presented in this study are available at http:/doi.org/10.34740/kaggle/dsv/4961781.

## References

[1] Epstein JI, Zelefsky MJ, Sjoberg DD, A contemporary prostate cancer grading system: A validated alternative to the Gleason score. Eur Urol. 2016;69(3):428–435. 10.1016/j.eururo.2015.06.046 26166626 PMC5002992

[2] Amaro A, Esposito AI, Gallina A, Validation of proposed prostate cancer biomarkers with gene expression data: A long road to travel. Cancer Metastasis Rev. 2014;33(2–3):657–671. 10.1007/s10555-013-9470-4 24477410 PMC4113682

[3] Mir MC, Li J, Klink JC, Kattan MW, Klein EA, Stephenson AJ. Optimal definition of biochemical recurrence after radical prostatectomy depends on pathologic risk factors: identifying candidates for early salvage therapy. Eur Urol. 2014;66(2):204–210. 10.1016/j.eururo.2013.08.022 24007712

[4] Sokoll LJ, Zhang Z, Chan DW, Do ultrasensitive prostate specific antigen measurements have a role in predicting long-term biochemical recurrence-free survival in men after radical prostatectomy? J Urol. 2016;195(2):330–336. 10.1016/j.juro.2015.08.080 26307160

[5] Madabhushi A Digital pathology image analysis: opportunities and challenges. Imaging Med. 2009;1(1):7–10. 10.2217/IIM.09.9 30147749 PMC6107089

[6] Niazi MKK, Parwani AV, Gurcan MN. Digital pathology and artificial intelligence. Lancet Oncol. 2019;20(5):e253–e261. 10.1016/S1470-2045(19)30154-8 31044723 PMC8711251

[7] Sparks R, Madabhushi A. Explicit shape descriptors: novel morphologic features for histopathology classification. Med Image Anal. 2013;17(8):997–1009. 10.1016/j.media.2013.06.002 23850744 PMC3811112

[8] Ali S, Veltri R, Epstein JI, Christudass C, Madabhushi A. Selective invocation of shape priors for deformable segmentation and morphologic classification of prostate cancer tissue microarrays. Comput Med Imaging Graph. 2015;41:3–13. 10.1016/j.compmedimag.2014.11.001 25466771 PMC4346384

[9] Lee G, Sparks R, Ali S, Co-occurring gland angularity in localized subgraphs: predicting biochemical recurrence in intermediate-risk prostate cancer patients. PLOS ONE. 2014;9(5):e97954. 10.1371/journal.pone.0097954 24875018 PMC4038543

[10] Sood A, Jeong W, Peabody JO, Hemal AK, Menon M. Robot-assisted radical prostatectomy: inching toward gold standard. Urol Clin North Am. 2014;41(4):473–484. 10.1016/j.ucl.2014.07.002 25306159

[11] Menon M, Hemal AK, VIP Team. Vattikuti Institute prostatectomy: A technique of robotic radical prostatectomy: experience in more than 1000 cases. J Endourol. 2004;18(7):611–619. 10.1089/end.2004.18.611. discussion 619. 15597646

[12] Shah V, Pohida T, Turkbey B, A method for correlating in vivo prostate magnetic resonance imaging and histopathology using individualized magnetic resonance -based molds. Rev Sci Instrum. 2009;80(10):104301. 10.1063/1.3242697 19895076 PMC2774342

[13] Cox RW. AFNI: software for analysis and visualization of functional magnetic resonance neuroimages. Comput Biomed Res. 1996;29(3):162–173. 10.1006/cbmr.1996.0014 8812068

[14] Hurrell SL, McGarry SD, Kaczmarowski A, Optimized b-value selection for the discrimination of prostate cancer grades, including the cribriform pattern, using diffusion weighted imaging. J Med Imaging (Bellingham). 2018;5(1):011004. 10.1117/1.jmi.5.1.011004 29098169 PMC5658575

[15] McGarry SD, Hurrell SL, Iczkowski KA, Radio-pathomic maps of epithelium and lumen density predict the location of high-grade prostate cancer. Int J Radiat Oncol Biol Phys. 2018;101(5):1179–1187. 10.1016/j.ijrobp.2018.04.044 29908785 PMC6190585

[16] McGarry SD, Bukowy JD, Iczkowski KA, Gleason probability maps: A radiomics tool for mapping prostate cancer likelihood in mri space. Tomography. 2019;5(1):127–134. 10.18383/j.tom.2018.00033 30854450 PMC6403022

[17] McGarry SD, Bukowy JD, Iczkowski KA, Radio-pathomic mapping model generated using annotations from five pathologists reliably distinguishes high-grade prostate cancer. J Med Imaging (Bellingham). 2020;7(5):054501. 10.1117/1.jmi.7.5.054501 32923510 PMC7479263

[18] Iczkowski KA, Torkko KC, Kotnis GR, Digital quantification of five high-grade prostate cancer patterns, including the cribriform pattern, and their association with adverse outcome. Am J Clin Pathol. 2011;136(1):98–107. 10.1309/AJCPZ7WBU9YXSJPE 21685037 PMC4656017

[19] Iczkowski KA, Paner GP, Van der Kwast T. The new realization about cribriform prostate cancer. Adv Anat Pathol. 2018;25(1):31–37. 10.1097/PAP.0000000000000168 28820750

[20] Kweldam CF, Wildhagen MF, Steyerberg EW, Bangma CH, Van Der Kwast TH, Van Leenders GJLH. Cribriform growth is highly predictive for postoperative metastasis and disease-specific death in Gleason score 7 prostate cancer. Mod Pathol. 2015;28(3):457–464. 10.1038/modpathol.2014.116 25189638

[21] Montironi R, Cimadamore A, Gasparrini S, Prostate cancer with cribriform morphology: diagnosis, aggressiveness, molecular pathology and possible relationships with intraductal carcinoma. Expert Rev Anticancer Ther. 2018;18(7):685–693. 10.1080/14737140.2018.1469406 29699428

[22] van der Slot MA, Hollemans E, den Bakker MA, Inter-observer variability of cribriform architecture and percent Gleason pattern 4 in prostate cancer: relation to clinical outcome. Virchows Arch. 2021;478(2):249–256. 10.1007/s00428-020-02902-9 32815034 PMC7969485

[23] Ruifrok AC, Johnston DA. Quantification of histochemical staining by color deconvolution. Anal Quant Cytol Histol. 2001;23(4):291–299. 11531144

[24] Storey JD. A direct approach to false discovery rates. J R Stat Soc B. 2002;64(3):479–498. 10.1111/1467-9868.00346

[25] Hollemans E, Verhoef EI, Bangma CH, Large cribriform growth pattern identifies ISUP grade 2 prostate cancer at high risk for recurrence and metastasis. Mod Pathol. 2019;32(1):139–146. 10.1038/s41379-018-0157-9 30349027 PMC6300553

[26] Cooperberg MR, Pasta DJ, Elkin EP, The University of California, San Francisco Cancer of the Prostate Risk Assessment score: a straightforward and reliable preoperative predictor of disease recurrence after radical prostatectomy. J Urol. 2005;173(6):1938–1942. 10.1097/01.ju.0000158155.33890.e7 15879786 PMC2948569

[27] Brajtbord JS, Leapman MS, Cooperberg MR. The CAPRA score at 10 years: contemporary perspectives and analysis of supporting studies. Eur Urol. 2017;71(5):705–709. 10.1016/j.eururo.2016.08.065 27616723

[28] Ozkan TA, Eruyar AT, Cebeci OO, Memik O, Ozcan L, Kuskonmaz I. Inter-observer variability in Gleason histological grading of prostate cancer. Scand J Urol. 2016;50(6):420–424. 10.1080/21681805.2016.1206619 27416104

[29] Leo P, Janowczyk A, Elliott R, Computer extracted gland features from H&E predicts prostate cancer recurrence comparably to a genomic companion diagnostic test: a large multi-site study. npj Precis Oncol. 2021;5(1):35. 10.1038/s41698-021-00174-3 33941830 PMC8093226

[30] Park J, Rho MJ, Moon HW, Dr. Answer Ai for prostate cancer: predicting biochemical recurrence following radical prostatectomy. Technol Cancer Res Treat. 2021;20:15330338211024660. 10.1177/15330338211024660 34180308 PMC8243093

