# Supplementary material for: Quantitative Histomorphometric Features of Prostate Cancer Predict Patients Who Biochemically Recur Following Prostatectomy
Source: Lab Invest. Author manuscript; Available in PMC 2024 Feb 16. (PMC10872376; doi:10.1016/j.labinv.2023.100269)
Supplement: Supplemental Doc [file NIHMS1951617-supplement-Supplemental_Doc.docx]

**Supplementary**

**
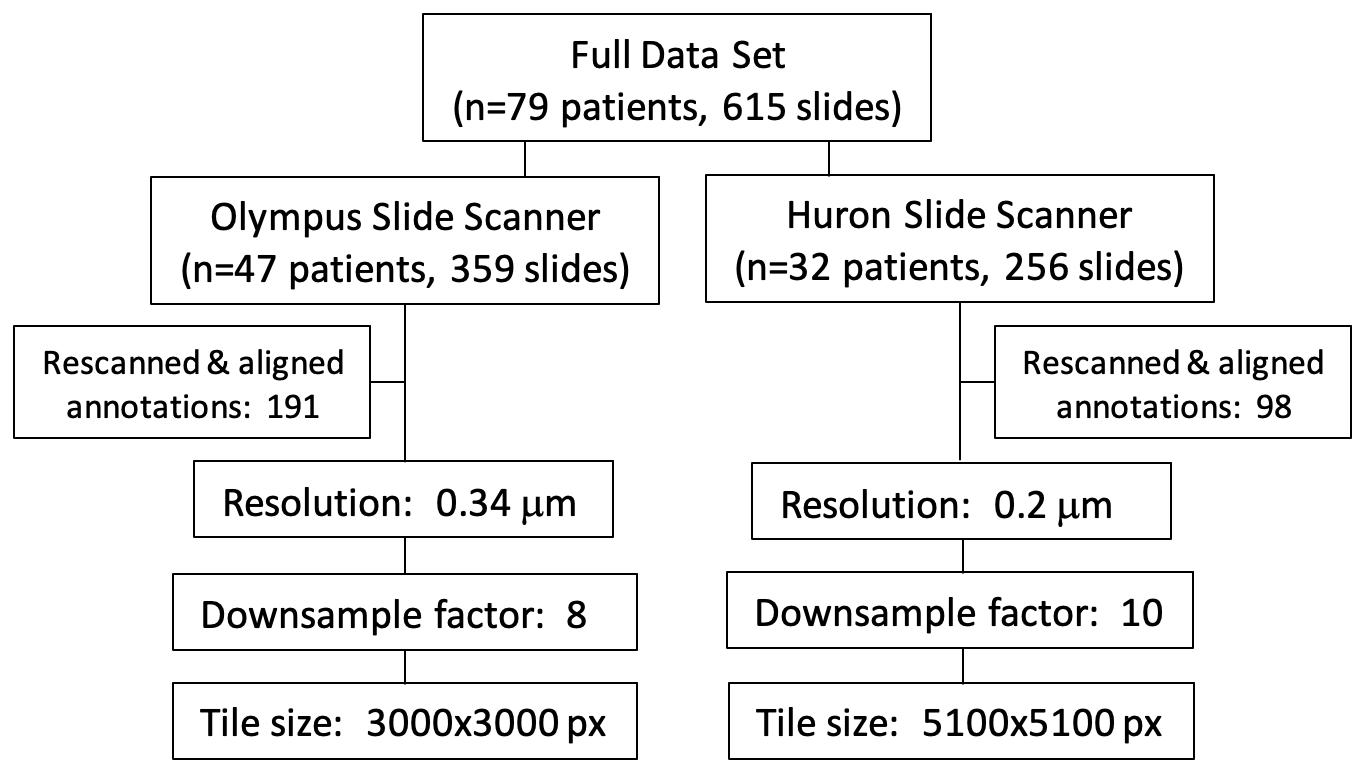
**

**Figure 1**. Flowchart of digital slide scanners and their respective properties.

**
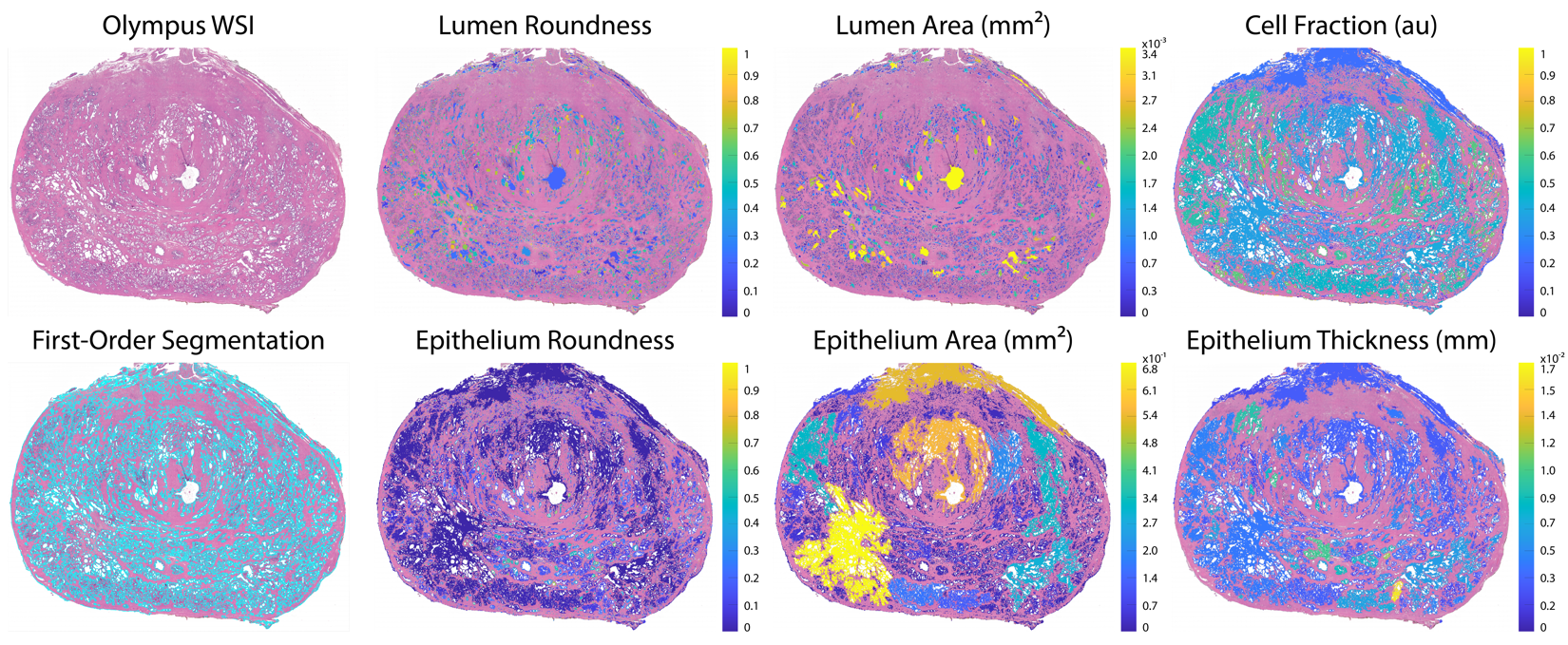
**

**Figure 2**. Feature segmentations performed on a whole slide image scanned using the Olympus slide scanner.

**­**

**­­**

**Table 1.** Logistic regression feature importance from clinical features, Grade Group only, and CAPRA only models. B-values are shown with standard error (S.E.) and degrees of freedom (d.f.).

| Group | Feature | B (S.E.) | d.f. | p-value |
| --- | --- | --- | --- | --- |
| Clinical Information | Age (years) | -0.08 (0.06) | 1 | 0.19 |
|  | pT3b (ref.) | 0 (0) | 5 | 0.98 |
|  | pT2 | 0.54 (41529.56) | 1 | 1.00 |
|  | pT2a | -16.8 (16906.06) | 1 | 1.00 |
|  | pT2b | -18.4 (25544.47) | 1 | 1.00 |
|  | pT2c | 0.75 (1.08) | 1 | 0.49 |
|  | pT3a | 0.95 (1.03) | 1 | 0.36 |
|  | Grade Group 5 (ref.) |  | 4 | 0.55 |
|  | Grade Group 1 | -19.98 (9.74E+3) | 1 | 1.00 |
|  | Grade Group 2 | 0.22 (0.99) | 1 | 0.82 |
|  | Grade Group 3 | 1.4 (1.02) | 1 | 0.17 |
|  | Grade Group 4 | 1.13 (1.71) | 1 | 0.51 |
|  | Baseline PSA (ng/mL) | 0 (0.06) | 1 | 0.96 |
|  | Tumor Ratio (au) | -2.03 (7.27) | 1 | 0.78 |
|  | Tumor Volume (mm3) | 0.02 (0.2) | 1 | 0.91 |
| Grade Group | Grade Group 5 (ref.) |  | 4 | 0.80 |
|  | Grade Group 1 | -20.64 (10742.02) | 1 | 1.00 |
|  | Grade Group 2 | -0.83 (0.74) | 1 | 0.27 |
|  | Grade Group 3 | -0.13 (0.88) | 1 | 0.88 |
|  | Grade Group 4 | -20.64 (28420.72) | 1 | 1.00 |
| CAPRA | CAPRA Score | 0.64 (0.20) | 1 | 0.00 |

**Table 2.** Logistic regression feature importance from combined pathomic and clinical feature models stratified by features calculated across all tiles and WSI. β-values are shown with standard error (S.E.) and degrees of freedom (d.f.).

| Group | Feature | β (S.E.) | d.f. | p-value |
| --- | --- | --- | --- | --- |
| Tile Pathomic Features with Clinical Information | Age (years) | -0.15 (0.09) | 1 | 0.11 |
|  | Surgery Stage pT3 (ref.) | 0 (0) | 5 | 0.29 |
|  | Surgery Stage pT1 | 25.88 (40192.97) | 1 | 1.00 |
|  | Surgery Stage pT2 | -18.72 (18246.4) | 1 | 1.00 |
|  | Grade Group 5 (ref.) | 4.33 (9.11) | 1 | 0.63 |
|  | Grade Group 1 | 0.17 (1.4) | 1 | 0.91 |
|  | Grade Group 2 | -6.39 (2.8) | 1 | 0.02 |
|  | Grade Group 3 | 0 (0) | 4 | 0.40 |
|  | Grade Group 4 | 45.41 (5992.26) | 1 | 0.99 |
|  | Baseline PSA (ng/mL) | 48.6 (5992.26) | 1 | 0.99 |
|  | Tumor Ratio (au) | 50.95 (5992.26) | 1 | 0.99 |
|  | Tumor Volume (mm^3^) | 27.66 (27791.69) | 1 | 1.00 |
|  | Stroma Area (mm^2^) | -0.1 (0.1) | 1 | 0.32 |
|  | Epithelial Area (mm^2^) | -42.86 (19.05) | 1 | 0.02 |
|  | Epithelial Size (mm^2^) | 2.01 (0.76) | 1 | 0.01 |
|  | Epithelial Roundness (au) | 37.13 (132.36) | 1 | 0.78 |
|  | Epithelial Wall Thickness (mm) | 143.68 (109.1) | 1 | 0.19 |
|  | Cell Fraction (au) | 57.06 (372.66) | 1 | 0.88 |
|  | Lumen Area (mm^2^) | 35.69 (72.25) | 1 | 0.62 |
|  | Lumen Roundness (au) | 8180.17 (10844.51) | 1 | 0.45 |
| WSI Pathomic Features with Clinical Information | Age | -0.15 (0.09) | 1 | 0.09 |
|  | pT3b (ref.) | 0 (0) | 5 | 0.51 |
|  | pT2 | 23.27 (40192.97) | 1 | 1.00 |
|  | pT2a | -18.69 (18423.62) | 1 | 1.00 |
|  | pT2b | 1.88 (2.38) | 1 | 0.43 |
|  | pT2c | 0.34 (1.43) | 1 | 0.81 |
|  | pT3a | -6.14 (3.3) | 1 | 0.06 |
|  | Grade Group 5 (ref.) | 0 (0) | 4 | 0.79 |
|  | Grade Group 1 | 32.41 (6245.59) | 1 | 1.00 |
|  | Grade Group 2 | 33.76 (6245.59) | 1 | 1.00 |
|  | Grade Group 3 | 34.8 (6245.59) | 1 | 1.00 |
|  | Grade Group 4 | 14.54 (28294.65) | 1 | 1.00 |
|  | Baseline PSA (ng/mL) | 0.08 (0.1) | 1 | 0.41 |
|  | Tumor Ratio (au) | -32.68 (15.38) | 1 | 0.03 |
|  | Tumor Volume (mm^3^) | 1.4 (0.63) | 1 | 0.03 |
|  | Stroma Area (mm^2^) | 132.89 (90.52) | 1 | 0.14 |
|  | Epithelial Area (mm^2^) | 81.08 (70.31) | 1 | 0.25 |
|  | Epithelial Size (mm^2^) | -0.03 (0.04) | 1 | 0.38 |
|  | Epithelial Roundness (au) | -7.97 (22.47) | 1 | 0.72 |
|  | Epithelial Wall Thickness (mm) | 4.71 (5.72) | 1 | 0.41 |
|  | Cell Fraction (au) | 9.11 (11.63) | 1 | 0.43 |
|  | Lumen Area (mm^2^) | 275.67 (171.02) | 1 | 0.11 |
|  | Lumen Roundness (au) | 8.87 (7.64) | 1 | 0.25 |
